# Technology-based health solutions for cancer caregivers to better shoulder the impact of COVID-19: a systematic review protocol

**DOI:** 10.1186/s13643-021-01592-x

**Published:** 2021-02-01

**Authors:** Zhaohui Su, Dean McDonnell, Bin Liang, Jennifer Kue, Xiaoshan Li, Sabina Šegalo, Shailesh Advani, Bertha E. Flores, Jing Wang

**Affiliations:** 1 School of Nursing, Center on Smart and Connected Health Technologies, Mays Cancer Center, UT Health San Antonio, San Antonio, TX 78229 USA; 2 Department of Humanities, Institute of Technology Carlow, Carlow, R93 V960, Ireland; 3 Department of Radiation Oncology, National Cancer Center/National Clinical Research Center for Cancer/Cancer Hospital, Chinese Academy of Medical Sciences and Peking Union Medical College, Beijing, 10010 China; 4 School of Nursing, Ohio State University, Columbus, USA; 5 Program of Public Relations and Advertising, Beijing Normal University-Hong Kong Baptist University United International College, Zhuhai, China; 6 Department of Microbiology, Faculty of Medicine, University of Sarajevo, 71000 Sarajevo, Bosnia and Herzegovina; 7 Terasaki Institute of Biomedical Innovation, Los Angeles, CA 21100 USA; 8 School of Nursing, UT Health San Antonio, San Antonio, TX 78229 USA

**Keywords:** COVID-19, Coronavirus, Technology-based interventions, Cancer caregivers, Cancer patients, Systematic review protocol, Telemedicine, Digital health solutions

## Abstract

**Background:**

Cancer patients are particularly vulnerable to COVID-19, partially owing to their compromised immune systems and curbed or cut cancer healthcare services caused by the pandemic. As a result, cancer caregivers may have to shoulder triple crises: the COVID-19 pandemic, pronounced healthcare needs from the patient, and elevated need for care from within. While technology-based health interventions have the potential to address unique challenges cancer caregivers face amid COVID-19, limited insights are available. Thus, to bridge this gap, we aim to identify technology-based interventions designed for cancer caregivers and report the characteristics and effects of these interventions concerning cancer caregivers' distinctive challenges amid COVID-19.

**Methods:**

A systematic search of the literature will be conducted in PubMed, PsycINFO, CINAHL, and Scopus from the database inception to the end of March 2021. Articles that center on technology-based interventions for cancer caregivers will be included in the review. The search strategy will be developed in consultation with an academic librarian who is experienced in systematic review studies. Titles, abstracts, and full-text articles will be screened against eligibility criteria developed a priori. The Preferred Reporting Items for Systematic Reviews and Meta-Analyses procedures will be followed for the reporting process.

**Conclusions:**

COVID-19 has upended cancer care as we know it. Findings of this study can shed light on evidence-based and practical solutions cancer caregivers can utilize to mitigate the unique challenges they face amid COVID-19. Furthermore, results of this study will also offer valuable insights for researchers who aim to develop interventions for cancer caregivers in the context of COVID-19. In addition, we also expect to be able to identify areas for improvement that need to be addressed in order for health experts to more adequately help cancer caregivers weather the storm of global health crises like COVID-19 and beyond.

**Systematic review registration:**

PROSPERO CRD42020196301

**Supplementary Information:**

The online version contains supplementary material available at 10.1186/s13643-021-01592-x.

## Background

A growing body of research is exploring the impact of COVID-19 on cancer care and management [1–3]. Already acknowledged as being infectious and deadly [4, 5], as of 8 January 22^nd^, 2021, there are approximately 96.2 million confirmed COVID-19 cases, among which, 2.06 million deaths have already occurred [6]. Through a retrospective analysis of 355 patients dying from the coronavirus, one in five of these patients had active cancer [7]. Individuals with cancer can experience underlying malignancy, treatment-induced immunosuppression, and possible comorbidity [8–10], and it has been shown that they are more likely to develop severe symptoms from COVID-19 [8, 9, 11]. Research also indicates that, compared to COVID-19 patients without cancer, COVID-19 patients with cancer are more likely to have higher risks in all severe outcomes (e.g., higher mortality rates) [8, 9]. Additional factors may further increase cancer patients' vulnerability to COVID-19, such as limited access to medical resources and cancer care, during this pandemic [12–14].

Due to medical resource rationing, many cancer care and treatment services were either canceled or indefinitely postponed during the early part of the COVID-19 pandemic [15, 16]. No longer having access to the healthcare services they were accustomed to or depended upon [11, 16], informal cancer caregivers may now be shouldering considerably more caregiver burden due to COVID-19. While the effects of this deprivation of access to cancer care on cancer patients are well discussed [17, 18], caregiving responsibilities influencing cancer caregivers’ health and well-being is less examined. Other than healthcare professionals in a caring role as a part of their work, an informal caregiver is generally offered unpaid or ill-compensated care to a family member or a friend, due to disease-centered or aging-related reasons. Pre-COVID-19 data show that caregivers shoulder approximately 70–89% of all care needed by patients in general [19]. Considering the interruptions COVID-19 exerts on cancer care and treatment, it is probable that cancer caregivers are shouldering even greater caregiving responsibilities for patients.

Cancer caregivers have been facing tremendous stressors during the COVID-19 pandemic. The range of issues resembles a triple crisis of (1) confronting the impact of the coronavirus outbreak, (2) shouldering pronounced care needs from the patient, and (3) coping with considerable needs for physical and psychological care from within. In other words, in addition to being forced to deal with a pandemic and patients’ pronounced cancer care needs discussed above, caregivers may also experience substantial physical and psychological health issues that require timely medical attention. Mounting evidence indicates that cancer caregivers often face considerable caregiver burden that can negatively impact their physical and psychosocial health [20–22]. In a review study, findings on 21,149 caregivers show that the prevalence of depression and anxiety is 42.30% and 46.55% in these caregivers, respectively [23]. It is important to note that blanket measures, such as lockdowns, self-isolation, and social distancing, can exert further pressure on cancer caregivers. Research suggests that social support from community members can lower anxiety and depression experienced by cancer caregivers; these supports are significantly limited due to social distancing recommendations [24].

Technology-based interventions refer to “the use of technology to manage or support health promotion strategies aiming to produce accessible and affordable health solutions to the target audience” [25]. Studies have shown that technologies (e.g., telehealth) may be beneficial to address issues cancer caregivers experienced during COVID-19; with some research identifying the potential improvement to health and well-being [26–28]. Technology-based interventions can offer greater accessibility to care for cancer caregivers that can be (1) delivered remotely without physical contacts between interventionists and the caregivers [29, 30], (2) received cost-effectively without the need for transportation [27], and accessed conveniently with self-paced learning [31, 32] of tailored content [33, 34]. In addressing the unique challenges cancer caregivers face amidst COVID-19, no research has identified technology-based health solutions for cancer patients that can address these needs, such as care needs, general healthcare needs, information and communication needs, and social support needs (see Table 1). Thus, to bridge this gap, this systematic review identifies the literature surrounding technology-based solutions for cancer caregivers that can mitigate challenges they face amid COVID-19.

**Table 1:** Cancer caregivers' unique needs associated with COVID-19

Cancer caregivers’ unique needs associated with COVID-19	
Unique challenges due to COVID-19	Need category
Due to cancer patients' canceled or delayed access to cancer care owing to heightened healthcare needs among COVID-19 patients, patients may need to rely more on caregivers for their care needs compared to their pre-COVID-19 normal.	Cancer care needs
As a result of negative impacts of COVID-19 and striking caregiver burden amid COVID-19, cancer caregivers may need healthcare services that can address issues associated with their physical and psychological health.	General healthcare needs
In addition to pronounced need for information on healthcare, due to the fear and uncertainty surround COVID-19, caregivers may need more information to help themselves as well as patients to cope with the impacts associated with COVID-19.	Information and communication needs
Protective measures against the spread of COVID-19 (e.g., lockdowns, self-isolation, and social distancing)	Social support needs

## Methods

### Study registration and protocol

This review was registered with the International Prospective Register of Systematic Reviews or PROSPERO (CRD42020196301). We will also follow the Preferred Reporting Items for Systematic Reviews and Meta-Analyses (PRISMA) in our research procedures [35] to further safeguard research rigor.

### Search strategy

The following databases will be searched for potential articles: PubMed, PsycINFO CINAHL, and Scopus. The search will be limited to original articles published in English from the database inception to the end of March 2021. Searches incorporated medical subject heading (MeSH) and keyword terms in three categories: cancer, caregivers, and technology platforms. Our search strategy will be developed in consultation with an academic librarian experienced in systematic review studies to ensure research rigor. Snowballing (manual searching reference lists) of included studies will be conducted to obtain additional eligible articles. Furthermore, reverse tracing potential eligible manuscripts that cited included papers will be administered via Google Scholar. An example search string is listed in Table 2.

**Table 2:** Example PubMed search string

Concept	Search string
Cancer	cancer*[MeSH] OR cancer*[TIAB] OR tumor*[MeSH] OR tumor*[TIAB] OR tumour*[MeSH] OR tumour*[TIAB] OR neoplasms[MeSH] OR neoplasms[TIAB]
Caregivers	caregiver*[MeSH] OR caregiver*[TIAB] OR famil*[MeSH] OR famil*[TIAB] OR spous*[MeSH] OR spous*[TIAB] OR dyad* [MeSH] dyad* [TIAB] OR partner*[MeSH] OR partner*[TIAB] OR couple*[MeSH] OR couple*[TIAB]
Technology-based interventions	“technology”[MeSH] OR “technology”[TIAB] OR “eHealth”[TIAB] OR “telemedicine”[MeSH] OR “telemedicine”[TIAB] OR “tele-medicine”[MeSH] OR “tele-medicine”[TIAB] OR “telehealth”[TIAB] OR “tele-health”[TIAB] OR “connected health”[TIAB] OR “digital health”[TIAB] OR “mHealth”[TIAB] OR “mobile health”[TIAB]

### Inclusion and exclusion criteria

In the context of this study, caregivers are defined as patients’ family or friends who may offer mostly long-term care to patients, often with little or no financial compensation of any form. This paper broadly defines interventions as stimuli or mechanisms that are aimed to produce changes in outcome variables (e.g., self-care abilities increased). Studies will be included if they are published in English, have relevant information on technology-based interventions for cancer caregiving, and with detailed inclusion criteria listed in Table 3. Ensuring data quality, comments, editorials, gray literature, and reviews will be excluded from the review. Overall, articles will be excluded if they (1) did not include a cancer caregiver population, (2) did not provide information on intervention, and (3) did not describe how technology is integrated into the intervention strategy.

**Table 3:** Study inclusion criteria

Data type	Inclusion criteria
Participants	Cancer caregivers (≥ 1 years old)
Language	English
Study type	Original journal article
Study design	Focus on technology-based intervention that aim to improve cancer caregivers’ health and wellbeing
Intervention	Technology-based interventions for cancer caregivers
Outcome	Report empirical findings on intervention outcomes

### Selection of studies and data extraction

Search results will be managed using Rayyan [36], a free web application that allows sorting and storing articles will be used to remove duplicate records and screen articles. Both two phrases of screening, title-abstract screening and full-text screening, will be conducted by two primary reviewers (ZS and XL) independently. Discrepancies will be solved by consensus, and when needed, with input from the rest of the research team. Data will be extracted independently by the reviewers (ZS and XL) based on the research aim and selection criteria adopted in this study. Specifically, for studies that met the inclusion criteria, the primary reviewers (ZS and XL) will extract the following information from the final included studies: study and participant characteristics (e.g., study aim), intervention characteristics (e.g., the use of technology in interventions), and details on study outcomes (e.g., intervention outcomes).

### Data synthesis and analysis

If eligible studies are found to be heterogeneous, we will conduct a narrative synthesis instead, as opposed to a meta-analysis, to summarize key insights from the data. A summary of the key information extracted will be utilized to synthesize the main research findings. Descriptive analysis will be performed on categorical variables. If there are enough similarities in eligible papers to be pooled, a meta-analysis will be conducted to obtain further insights from the data. The Cochrane's *Q* test and *I*^*2*^ test will be adopted to calculate heterogeneity within studies—*I*^*2*^ values of 25%, 50%, and 75% suggest low, medium, and high heterogeneity, respectively [37]. If much heterogeneity is present, random-effect models will be adopted (as opposed to fixed-effect models), as these models are more robust in detecting large variations in studies [38]. Review manager 5.3 (Cochrane Collaboration software) will be utilized to conduct the random effect model. Visual inspection of funnel plots will be used to evaluate publication bias if needed. If enough data are available for meaningful investigation (e.g., theoretically or clinically meaningful), subgroup analyses will be conducted for diverse types of cancer types, stages, interventions, follow-up duration, caregiver types, and country. A sensitivity analysis will be conducted by sequentially removing one study periodically and reanalyzing the data to evaluate the impact of individual studies on overall outcomes. This process will allow potentially methodologically flawed research included in the review; a study will be considered acceptable if it affects the meta-estimate of less than ± 5%.

### Risk of bias and quality of evidence

The Cochrane Collaboration evaluation framework will be utilized to investigate the risk of bias of articles that met the inclusion criteria, including selection, performance, detection, attrition, reporting, and other biases [39]. The framework has seven domains: random sequence generation, allocation concealment, blinding of participants and personnel, blinding of outcome assessment, incomplete outcome data, selective reporting, and any other source of bias. Two primary reviewers (ZS and XL) will evaluate the included articles’ quality independently, and give the articles a score (high, medium, or low) based on results of the evaluation. These reviewers will also independently adopt the Grading of Recommendations, Assessment, Development, and Evaluation (GRADE) framework to assess the overall quality of evidence of the eligible articles. The GRADE guideline has five domains: risk of bias, indirectness, inconsistency, imprecision, and publication bias [40]. Any discrepancies will be resolved via group discussions till a consensus is reached.

## Discussion

COVID-19 has upended cancer care as we know it [41–45]. Evidence shows that pandemics of COVID-19’s scale could be particularly deadly to vulnerable populations such as cancer patients [2, 3, 8–10, 46]. Furthermore, COVID-19 prevention mechanisms, such as lockdowns, self-isolation, and social distancing measures, as well as COVID-19-induced medical resources rationing, have curbed or cut cancer patients and their caregivers’ access to traditional healthcare services [47–50]. Not to mention chaos and consequences associated with pandemic information overload, COVID-19 infodemics, and dysfunctional vaccine dissemination may further compound cancer patients and their caregivers’ interaction with the ever-increasingly overstretched and overstressed healthcare systems [51–53]. As a result, cancer caregivers often have to step up to address patients’ healthcare needs and wants [42, 54–57], which, in turn, could exert substantial mental and physical stress on informal caregivers, above and beyond COVID-19-related burdens the general public shoulders daily [20–22]. Technology-based health solutions can bypass spatial distancing constraints caused by COVID-19 and have the ability to address unique challenges cancer patients, and their caregivers face amid COVID-19 [58–61].

As the frontline physician among us observed, the most challenging cancer care component during COVID-19 is how to resume cancer treatment for patients [1]. Because the pandemic has caused severe limitations to access to cancer care and availability of transportation, across the world, many patients, even in severe conditions, had to suspend their treatment plans [1, 56, 62]. Take Chinese cancer patients, for instance. The pandemic occurred during the Chinese traditional spring festival. As a result, a large number of cancer patients traveled home with their caregivers for their extended-family reunion. However, due to the outbreak [63], after the spring festival, most of them were under lockdown at their hometown or somewhere in between, without access to critical cancer care and treatment options. Even among patients and caregivers who managed to rush back to the hospital for their treatments, they had to be self-quarantined for 14 days and then undergo a series of tests. Technologies, such as the “Health Code”, a digital color-coded health system that allows the governments and health agencies to track cell phone location to better determine individuals’ whereabouts (i.e., whether they have recently traveled to places witnessed severe COVID-19 outbreaks) [64], undoubtedly have helped expedite the information processing speed, and saved valuable time these patients and caregivers desperately needed.

However, while useful insights are available in the literature, there is a dearth of research that can shed light on evidence-based and practical health solutions cancer caregivers can use to address and alleviate unique challenges they face during COVID-19 or any future disease pandemics. Therefore, to bridge this gap, we aim to identify technology-based interventions designed for cancer caregivers and report the characteristics and effects of these interventions concerning the distinctive challenges cancer caregivers face amid COVID-19. Additionally, this paper will present practical insights into the diverse intervention approaches that can help deliver digital health solutions for cancer caregivers amid and beyond COVID-19. Furthermore, this study’s results can also offer valuable insights for researchers who aim to develop interventions for cancer caregivers in the context of COVID-19. In addition, it is also expected to identify areas for improvement that need to be addressed in order for health experts to more adequately help cancer caregivers weather the storm of global health crises like COVID-19 and beyond.

## Supplementary Information

**Additional file 1:.** PRISMA-P checklist

## Data Availability

None

## Abbreviations

COVID-19: Coronavirus disease 2019
PRISMA: Preferred Reporting Items for Systematic Reviews and Meta-Analyses
PROSPERO: International Prospective Register of Systematic Reviews
